# Acute kidney disease following COVID-19 vaccination: a single-center retrospective study

**DOI:** 10.3389/fmed.2023.1189243

**Published:** 2023-05-22

**Authors:** Chien-Chou Chen, Sung-Sen Yang, Yu-Juei Hsu, Chih-Chien Sung, Pauling Chu, Chia-Chao Wu, Shun-Neng Hsu, Han-En Wang, Ding-Jie Lee, Shih-Hua Lin

**Affiliations:** Division of Nephrology, Department of Medicine, National Defense Medical Center, Tri-Service General Hospital, Taipei, Taiwan

**Keywords:** acute kidney disease (AKD), chronic kidney disease, COVID-19, glomerulonephritis (GN), Naranjo score, vaccination

## Abstract

**Background:**

Rare cases of *de novo* or relapsed kidney diseases associated with vaccination against coronavirus disease 2019 (COVID-19) have been increasingly reported. The aim of this study was to report the incidence, etiologies, and outcomes of acute kidney disease (AKD) following COVID-19 vaccination.

**Methods:**

This retrospective study extracted cases from renal registry of a single medical center from 1 March 2021 to 30 April 2022, prior to the significant surge in cases of the Omicron variant of COVID-19 infection in Taiwan. Adult patients who developed AKD after COVID-19 vaccination were included. We utilized the Naranjo score as a causality assessment tool for adverse vaccination reactions and charts review by peer nephrologists to exclude other causes. The etiologies, characteristics, and outcomes of AKD were examined.

**Results:**

Twenty-seven patients (aged 23 to 80 years) with AKD were identified from 1,897 vaccines (estimated rate of 13.6 per 1000 patient-years within the renal registry). A majority (77.8%) of vaccine received messenger RNA-based regimens. Their median (IQR) Naranjo score was 8 (6-9) points, while 14 of them (51.9%) had a definite probability (Naranjo score ≥ 9). The etiologies of AKD included glomerular disease (*n* = 16) consisting of seven IgA nephropathy, four anti-neutrophil cytoplasmic antibodies-associated glomerulonephritis (AAN), three membranous glomerulonephritis, two minimal change diseases, and chronic kidney disease (CKD) with acute deterioration (*n* = 11). Extra-renal manifestations were found in four patients. Over a median (IQR) follow-up period of 42 (36.5–49.5) weeks, six patients progressed to end-stage kidney disease (ESKD).

**Conclusion:**

Besides glomerulonephritis (GN), the occurrence of AKD following COVID-19 vaccination may be more concerning in high-risk CKD patients receiving multiple doses. Patients with the development of *de novo* AAN, concurrent extra-renal manifestations, or pre-existing moderate to severe CKD may exhibit poorer kidney prognosis.

## Introduction

In response to the severe acute respiratory syndrome coronavirus 2 (SARS-CoV-2) pandemic, vaccination against the coronavirus disease of 2019 (COVID-19) has been rapidly deployed under emergency use authorization (EUA) (1). Before the emergence of omicron variants in 2022, two types of vaccination against COVID-19 were approved, based on messenger RNA (mRNA) via lipid nanoparticles such as BNT162b2 (Pfizer-BioNTech)

and mRNA-1273 (Moderna) (2, 3), viral vectors such as AZD1222 (Oxford-AstraZeneca), Ad26.COV2.S (Janssen), and Gam-COVID-Vac (Sputnik V) (4–6). These COVID-19 vaccines can trigger and enhance innate and adaptive immune response with the activation of neutrophils, T cells, and B cells (7, 8). With the strengthened immunity, rare serious side effects have been increasingly reported after COVID-19 vaccination. These included immune-mediated myocarditis, vaccine-induced immune thrombotic thrombocytopenia (VITT), and various neurological complications such as seizures, Guillain–Barré syndrome, transverse myelitis, and Bell's palsy (9–11).

In the kidney, growing literature had reinforced the linkage between COVID-19 vaccination and the rapid development of *de novo* or relapsed glomerulonephritis (GN) (12, 13). To date, the establishment of COVID-19 vaccination-related kidney diseases mostly depended on temporal association with the exclusion of other clear causes due to the lack of mechanistic proof for confirmation. Hence, there is a discrepancy in the research regarding the link between COVID-19 vaccination and the development of acute kidney disease (AKD) (14). One large retrospective study from the nationwide institute of pathology did not find a higher incidence of GN during the COVID-19 vaccination campaign (15), but a separate study from a nationwide GN registry indicated an increased risk of relapse with a second or third dose of the COVID-19 vaccine, especially the mRNA type (16). Given these diverse results, it is crucial to utilize logical and analytical tools to acquire a comprehensive evaluation of adverse vaccine events. The Naranjo score as a causality assessment tool validated to determine the likelihood of an adverse drug reaction has been used to evaluate the relationship between adverse vaccine events and vaccines (17, 18). Nevertheless, its application has not yet extended to AKD and COVID-19 vaccines. Moreover, the impact of COVID-19 vaccination on accelerating chronic kidney disease (CKD) progression was still less appreciated.

In this study, we first utilized the Naranjo score as a probability measurement tool for adverse vaccination reactions and then chart review by peer nephrologists to exclude other causes at a renal registry of a single medical center in Taiwan prior to the severe COVID-19 pandemic outbreak (19). The etiologies, characteristics, and outcomes of COVID-19 vaccination-related AKD were also examined.

## Materials and methods

### Patient selection

**Figure 1:**
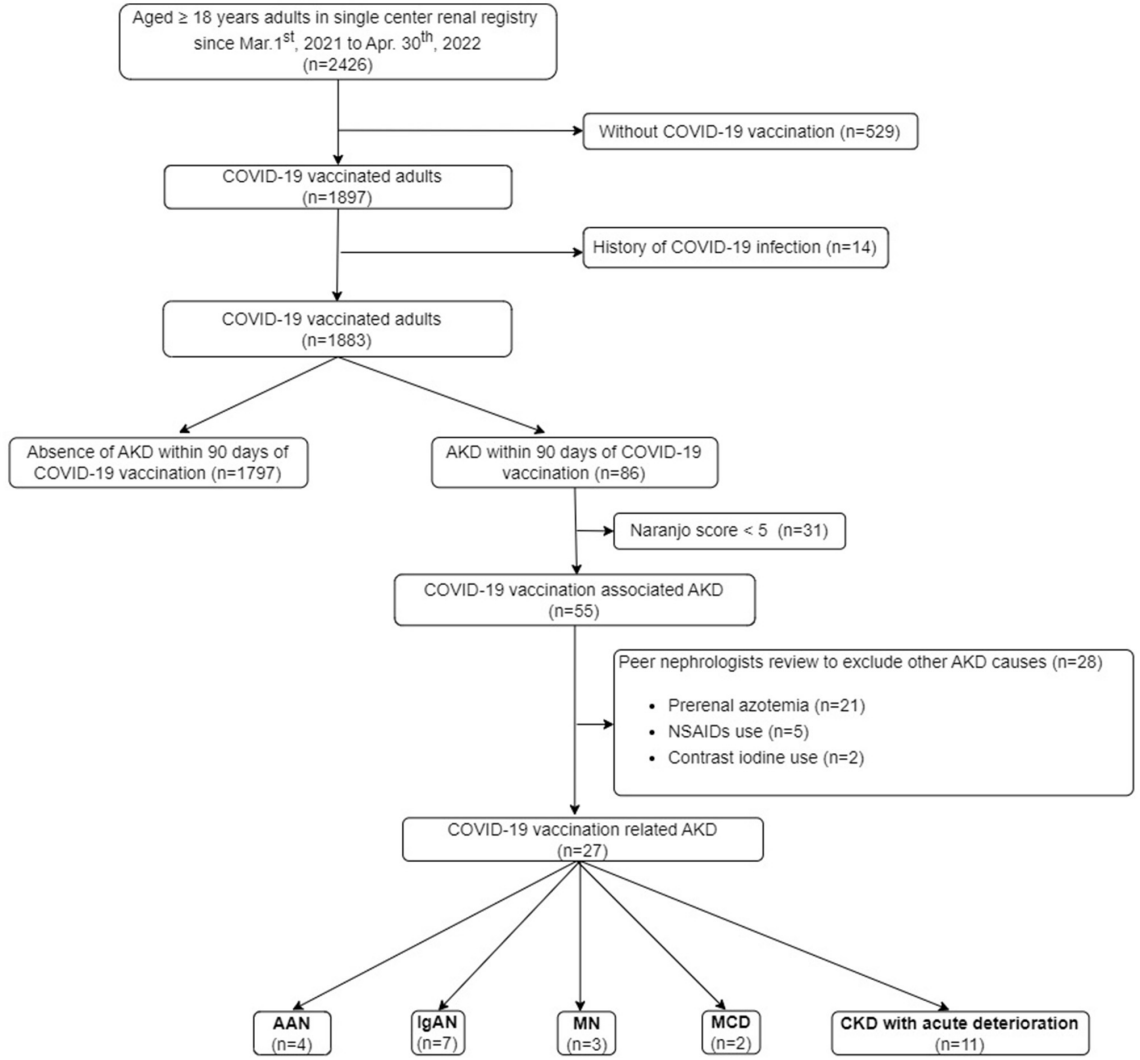
Flowchart of the study. AAN, anti-neutrophil cytoplasmic antibodies-associated glomerulonephritis; AKD, acute kidney disease; KTR, kidney transplant recipient; CKD, chronic kidney disease; COVID-19, coronavirus disease 2019; ESKD, end-stage kidney disease; IgAN, immunoglobulin A nephropathy; MCD, minimal change disease; MN, membranous nephropathy; NSAIDs, non-steroidal anti-inflammatory drugs; RRT, renal replacement therapy.

The study was approved by the Institutional Review Board (IRB) of Tri-service General Hospital, Taipei, Taiwan (IRB No. B202205155). The data of the study were based on an institutional clinical database, collected by Tri-service General Hospital Renal Registry (TSGHRR). The TSGHRR consisted of 2,426 adult patients with either a current or past occurrence of AKI or CKD, but not yet on renal replacement therapy (RRT) from both inpatient and outpatient services. In the TSGHRR, adult patients would regularly receive blood and urine examinations every 3 months. If any uncommon symptoms developed, further additional investigation will be arranged. The algorithm for the selection of AKD related to COVID-19 vaccination is shown in Figure 1. The definition of COVID-19 vaccination is based on the administration of two main approved types of vaccines against COVID-19 in Taiwan, including messenger RNA (mRNA) via lipid nanoparticles such as BNT162b2 (Pfizer-BioNTech) and mRNA-1273 (Moderna), viral vectors such as AZD1222 (Oxford-AstraZeneca), Ad26.COV2.S (Janssen). The adult patients aged 18 years or more presenting with AKD within 90 days after COVID-19 vaccination from 1 March 2021 to 30 April 2022 (before the significant surge in cases of the Omicron variant of COVID-19 in Taiwan) were preliminarily enrolled. Patients who had a confirmed history of COVID-19 infection through data from Taiwan's National Health Insurance MediCloud System (NHI MediCloud System) were excluded.

In order to objectively adjudicate the cause of AKD linked to vaccination for each case, Naranjo score, a causality assessment tool to determine the likelihood of an adverse drug reaction was utilized (17). The physicians computed the Naranjo score for each patient who experienced AKD within 90 days of receiving the COVID-19 vaccine. Patients with a Naranjo score of <5 (doubtful or possible probability) were excluded. Furthermore, the manual review of every chart by three peer nephrologists would also be performed to exclude other identifiable causes of AKD.

Acute kidney disease was defined as acute kidney injury (AKI) with structural damage lasting for over 7 days and persisting not yet over 90 days according to Kidney Disease Improving Global Outcomes (KDIGO) criteria (20). AKD should be also more suitable than AKI in a retrospective analysis since patients might have non-specific prodromal symptoms of kidney injury but had not been medically evaluated within 1 week (14). The term “relapsed” kidney disease was defined as recurrent biopsy-proven nephropathy that had never been present prior to vaccination. Based on the definition of “*de novo*” kidney disease, indeed, an undetected subclinical disease could have been uncovered after the trigger of vaccination. CKD with acute deterioration was defined as the accelerated decline of renal function without any identifiable etiologies of AKD but temporal associated with vaccination in patients with the underlying CKD, stage III (estimated glomerular filtration rate, eGFR< 60 mL/min/1.73m^2^) or more (21).

### Study procedures

The included patients were classified according to the etiologies of AKD. Parameters including demographics, vaccine dose and type, temporality of vaccine to the onset of symptoms, laboratory and kidney biopsy data, medical history, treatments, and clinical follow-up were collected. Data from these subjects were extracted from clinical databases by electronic medical records (EMRs). The values of the estimated eGFR were calculated using the renal disease equation (IDMS-MDRD) (22). Hematuria was defined as the coexistence of ≥1+ heme by dipstick and ≥25 urine red blood cells (URBCs) per high power field. The timing of the baseline serum creatinine (SCr) and urine protein to creatinine ratio (UPCR) was recognized as the latest data before the first dose of COVID-19 vaccination. The onset time among SCr, urine analysis, UPCR, lipid profiles, and serologic exams was determined within 48 h. To describe the relationship between time course, presentation, management, and outcome of AKD after COVID-19 vaccination, some representative cases were illustrated.

### Statistical analysis

Descriptive statistics were used to characterize subjects, etiologies, and laboratory data as the sample size is relatively small. We reported continuous data with median and interquartile ranges. Categorical data were found with numbers and percentages. Paired Student's *t-*test was compared in the patients with acute deteriorated CKD prior to and after COVID-19 vaccination.

## Results

### Incidence of vaccination-related AKD

As shown in Figure 1, 78.2% (1,897 out of 2,426) of the adult patients in our renal registry had been vaccinated against COVID-19. There were 86 patients developing AKD within 90 days after COVID-19 vaccination. After the exclusion of the patients with Naranjo score < 5 (*n* = 31) and other causes of AKD recognized by peer nephrologists (*n* = 28), 27 patients of COVID-19-associated AKD (estimated rate of 13.6 per 1,000 patient-years within the renal registry) were identified.

### Naranjo probability score

Their median (IQR) Naranjo score was 8 (6–9). Among the 27 cases of vaccination-related AKD in this study, we found that 14 patients who scored 9 points or more on the Naranjo score (definite probability) and seven patients scoring 5–8 points (probable causality) had received two or more doses of COVID-19 vaccinations. The remaining six patients (four GN and two CKD with acute deterioration) who had received only one dose of COVID-19 vaccination had a Naranjo score of 5–6 points.

### Types and doses of COVID-19 vaccination

As shown in Table 1, the most common type of COVID-19 vaccine was the mRNA (23 of 27, 85.2%). Twelve of 16 (75%) patients who developed GN and all patients with CKD with acute deterioration received mRNA vaccination. Regarding the doses of vaccination, AKD occurred after the first dose and both the first and second dose mRNA vaccination in 16 (16 of 23, 69.6%) and seven (7 of 23, 30.4%) patients, respectively. Eight of 27 (29.6%) patients receiving adenovectors vaccination developed AKD in one after the first dose and seven after both the first and second doses. Three developed AKD after the heterologous booster doses of vaccination (Cases 8, 18, and 25), and the other three after the homologous booster doses (Cases 5, 21, and 24).

**Table 1:** Characteristics and outcomes of COVID-19 vaccination related AKD.

**No.**	**Naranjo score**	**Sex/ Age**	**Underlying disease**	**Presenting symptoms**	***De novo*/relapsed**	**Kidney Bx**	**Onset time (wk)**	**Accumulated doses**	Vaccine profile (1^st^/2^nd^/3^rd^)^*^	**Baseline SCr (mg/dL)**	**Baseline UPCR (mg/g)**	**Laboratory during presentation**					**Laboratory during last follow-up**		
												**SCr (mg/dL)**	**UPCR (mg/g)**	**URBC**	**Serology finding**	**SAlb (g/dL)**	**F/U time(wk)**	**SCr (mg/dL)**	**UPCR (mg/g)**
**GN- AAN**																			
1	9 (Definite)	M/53	Healthy	Hematuria	*De novo*	Vasculitis w/fibrocellular crescent GN	9	2	**AZ**/ AZ/-	1.02	NA	10.7	3,996	4+	Anti-MPO	3.6	53	2.7	2,855
2	9 (Definite)	M/79	Prostate cancer	Hematuria	*De novo*	Vasculitis w/fibrous crescent GN	17	2	**Mod**/ Mod/-	1.1	128	11.2	4,017	4+	Anti-MPO	3.7	58	5.6	5,372
3	6 (Probable)	F/70	UTI	Hematuria Hemoptysis	*De novo* (w/pul hemorrhage)	Vasculitis w/cellular crescent GN	3	1	Mod/-/-	1	524	6.5	4,384	4+	Anti-MPO	3.5	59	2.9	2,260
4	6 (Probable)	M/49	CKD	Hematuria	*De novo* (w/myopericarditis)	Proliferative GN w/marked glomerulosclerosis & fibrous crescent	6	1	Mod/ -/ -	1.2	NA	13.2	10,050	4+	Anti-MPO	3.2	36	ESKD	NA
**GN- IgAN**																			
5	9 (Definite)	F/34	IgAN	Edema	Relapsed	NA	11	3	**Mod**/ **Mod**/ Mod	1.3	960	1.6	1,128	Negative	Unremarkable	4.3	45	1.5	973
6	9 (Definite)	F/27	IgAN	Hematuria	Relapsed	NA	1	2	AZ**/ AZ**/-	0.7	118	0.9	1,370	4+	ANA	4.2	42	0.8	947
7	9 (Definite)	M/23	IgAN	Hematuria Edema	Relapsed	NA	6	2	**AZ**/ AZ/-	0.8	339	3.2	759	1+	NA	3.5	36	1.4	188
8	9 (Definite)	F/29	Healthy	Hematuria	*De novo*	IgA nephropathy (2+IgA, 1+C3 gr mes)	1	3	**AZ**/ **AZ**/ Mod	0.7	NA	0.7	402	4+	Unremarkable	5	33	0.7	285
9	8 (Definite)	F/31	Healthy	Hematuria	*De novo*	IgA nephropathy (2+IgA and C3 gr mes)	1	2	**Mod**/ Mod/-	1	NA	1.8	2,963	3+	Unremarkable	3.9	34	1.2	860
10	6 (Probable)	M/24	IgAN	Hematuria	Relapsed	NA	1	1	Mod /-	1.2	392	1.7	1,226	4+	Unremarkable	3	37	1.5	252
11	5 (Probable)	F/76	HCVD	Hematuria	*De novo*	IgA nephropathy (3+IgA, 2+ C3 gr mes)	1	1	Pfizer/-	0.7	NA	2.8	1,396	2+	Unremarkable	3.9	44	3.3	3,379
**GN- MN**																			
12	6 (Probable)	M/66	Gout	Edema	*De novo*	PLA2R positive MN	4	2	Mod /Mod/-	1	NA	1	9,155	Negative	Anti-PLA2R	2.4	46	1.4	6,443
13	9 (Definite)	M/66	GB stone	Edema	*De novo*	PLA2R positive MN	20	2	**Mod**/ Mod/-	1.1	NA	1.3	6,474	Negative	ANA Anti-PLA2R	2.6	63	1.5	5,895
14	9 (Definite)	M/50	MN	Edema	Relapsed	NA	4	2	**Pfizer**/ Pfizer/-	1	2481	1.1	4,010	NA	Unremarkable	2.1	42	1.6	7,837
15	6 (Probable)	M/48	MCD	Foamy urine Edema	Relapsed	NA	7	2	Pfizer/ Pfizer/-	1	28	1.2	24,840	Negative	Unremarkable	2	43	1.0	58
16	5 (Probable)	F/56	MCD	Foamy urine Edema	*De novo*	MCD	6	2	AZ/ Pfizer/ -	0.7	Negative	1	10,050	Negative	Unremarkable	2.3	41	0.7	42
**CKD with acute deterioration**																			
17	9 (Definite)	F/47	KTR for 10 yrs	Foamy urine Edema	NA	NA	3	3	AZ/ **AZ**/ **Mod**	1	735	1.5	1,730	Negative	Unremarkable	4.4	34	1.3	1,024
18	9 (Definite)	M/32	KTR for 2 yrs	Edema	NA (w/myopericarditis)	NA	14	3	**AZ**/ **AZ**/ Mod	2.8	1,147	5.3	838	Negative	Unremarkable	3.7	42	4.6	377
19	9 (Definite)	M/80	DM	Oliguria	NA	NA	2	2	Mod/ **Mod**/-	1.3	69	8.5	11,105	4+	Unremarkable	2.8	30	1.8	338
20	8 (Probable)	M/74	DM, CAD	Edema	NA	NA	1	2	Mod/ Mod/ -	2.6	42	5	5,986	1+	NA	3.8	44	ESKD	NA
21	8 (Probable)	F/67	DM	Edema	NA	NA	5	3	Mod/Mod/ **Mod**	1.2	34	1.8	644	Negative	Unremarkable	4.1	32	2.0	552
22	6 (Probable)	M/58	DM	Edema	NA	NA	10	1	Mod/ -/ -	2.4	390	6.2	19,400	Negative	Unremarkable	2.4	44	ESKD	NA
23	9 (Definite)	M/59	HTN	Edema	NA	NA	2	2	Mod/ **Mod**/ -	4.6	1,455	11.7	4,612	Negative	NA	3.7	38	ESKD	NA
24	8 (Probable)	M/68	SS	Edema	NA	NA	6	3	Mod/**Mod**/ Mod	1.5	336	2.1	2,349	Negative	Anti-Ro	2.4	38	1.9	6,333
25	9 (Definite)	F/64	SS	Edema	NA	NA	2	3	AZ/ **AZ**/ **Mod**	3.7	3,956	8.5	4,195	Negative	ANA Anti-Ro	3.8	56	ESKD	NA
26	6 (Probable)	M/47	CKD	Edema	NA	NA	2	2	Pfizer/ Pfizer/ -	5.1	1,074	7.7	3,949	Negative	NA	4.4	58	ESKD	NA
27	6 (Probable)	F/46	CKD	Headache Nausea	NA	NA	4	1	Pfizer/-/-	1.3	36	1.9	206	Negative	Unremarkable	4.5	45	2.8	540

AAN, Anti-Neutrophil Cytoplasmic Antibodies-associated glomerulonephritis; ANA, antinuclear antibodies; anti-MPO, anti-myeloperoxidase; AZ, AstraZeneca; Pfizer, Pfizer-BioNTech; Bx, biopsy; CAD, coronary artery disease; CKD, chronic kidney disease; COVID-19, coronavirus disease 2019; DM, diabetes mellitus; F, female; GN, glomerulonephritis; gr mes, granular mesangial; HCVD, hypertensive cardiovascular disease; HPF, high power film; IgAN, Immunoglobulin A Nephropathy; KTR, kidney transplant recipient; M, male; MCD, minimal change disease; MN, Membranous Nephropathy; Mod, Moderna; NA, not available/not applicable; No., number; PLA2R, phospholipase A2 receptor; pul, pulmonary; SAlb, serum albumin; SCr, serum creatinine; SS, Sjogren's syndrome; UPCR, urine protein to creatinine ratio; URBC, urine red blood cells; UTI, urinary tract infection; wk, week; 4+: >100/HPF, 3+: 75–100/HPF, 2+: 50–75/HPF, 1+: 25–50/HPF, negative: 0–25/HPF. ^*^The bold font in vaccination profile implied the onset vaccine dose, and the underline font implied the timing of represented data.

### Etiologies, characteristics, and outcome of vaccination-related AKD

The etiologies of AKD included GN (*n* = 16, 59.3%) and CKD with acute deterioration (*n* = 11, 40.7%) with a median (IQR) follow-up period of 42 (36.5–49.5) weeks (Table 1).

### GN

They consisted of IgA nephropathy (IgAN) (*n* = 7, 25.9%), AAN (*n* = 4, 14.8%), membranous glomerulonephritis (MN) (*n* = 3, 11.1%), and minimal change disease (MCD) (*n* = 2, 6.9%). Ten of 16 (62.5%) were *de novo* with biopsy-proved diagnosis and the others were relapsing. Their median age was 49.5 years (range, 23–79 years) and exhibited relatively normal median (IQR) SCr of 1 (0.78–1.1) mg/dL prior to vaccination.

**Figure 2:**
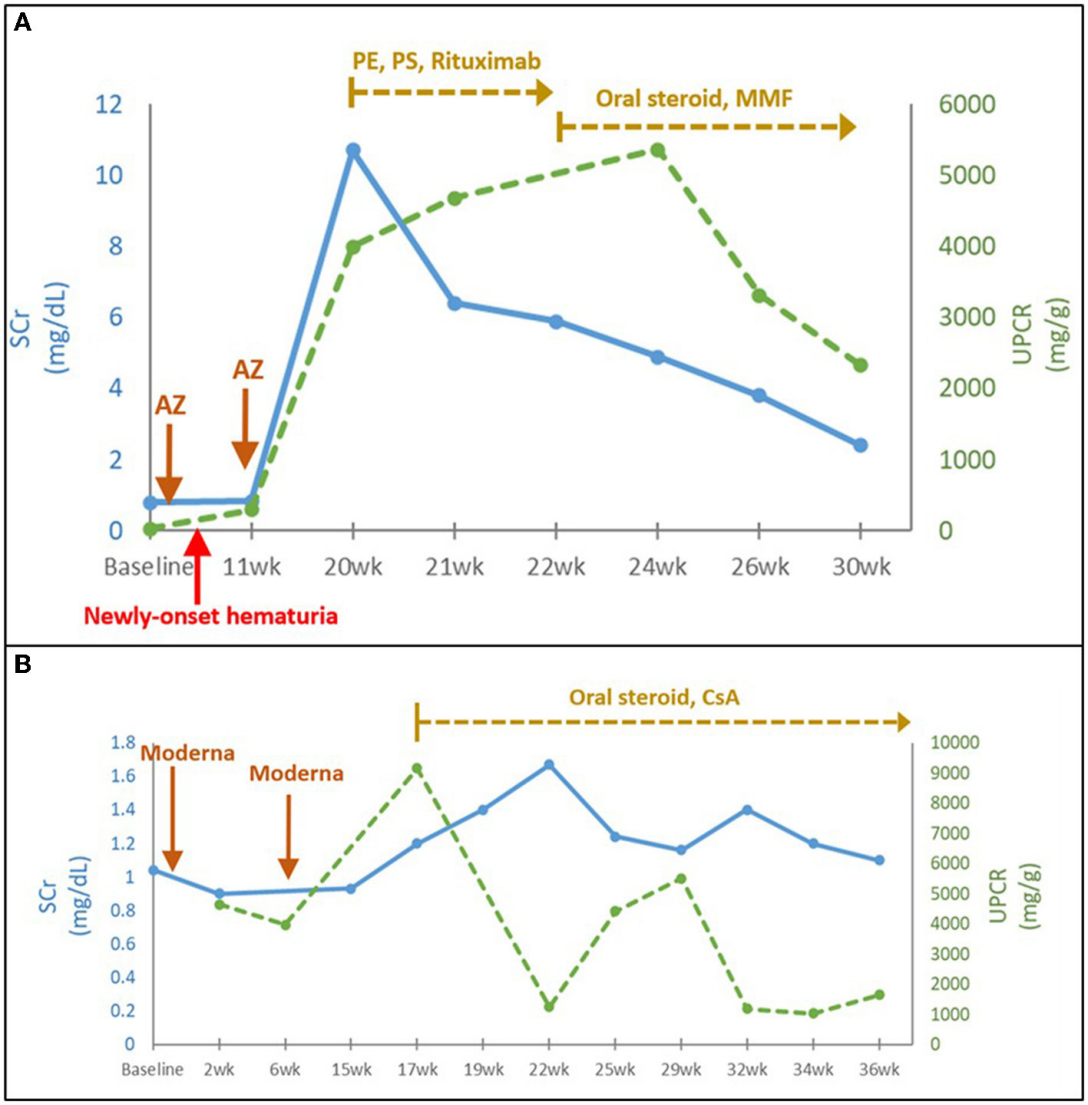
**(A)** Time course of AAN following COVID-19 vaccination (Case 1). **(B)** Time course of MN following COVID-19 vaccination (Case 12). AZ, AstraZeneca; CsA, cyclosporine; eGFR, estimated glomerular filtration rate; P.E., plasma exchange; P.S., pulse steroid; SCr, serum creatinine; UPCR, urine protein to creatinine ratio; wk., week.

Four patients with *de novo* biopsy-proven AAN exhibited severe hematuria, nephrotic proteinuria with median (IQR) UPCR of 4,201 (4,012–5,801) mg/g, and acute renal failure with median (IQR) SCr level from baseline 1.06 (1.0–1.1) to 11.0 (9.7–11.7) mg/dL) after COVID-19 vaccination. Fibrocellular and cellular crescents were predominantly found in kidney biopsies from two AAN patients with shorter onset duration (9 and 3 weeks between the vaccination and the onset in Cases 1 and 3, respectively). However, fibrous crescents were more prominent in another two AAN patients with longer onset duration (17 and 6 weeks between the vaccination and the onset in Cases 2 and 4, respectively). They all received aggressive management including pulse steroid therapy, emergent plasma exchange, intravenous immunoglobulin (IVIG), and anti-CD20 (Rituximab) treatment (Figure 2A). One patient with severe myopericarditis rapidly progressed to ESKD (Table 1, Case 4).

Seven IgAN patients including three *de novo*, biopsy-proven and four flared ones were relatively younger median (IQR) age of 29 (25.5–32.5) years. Most patients (six of seven, 85.7%) exhibited a rapid onset of gross hematuria after vaccination, accompanied by AKI with median (IQR) SCr from 0.8 (0.7–1.1) to 1.7 (1.3–2.3) mg/dL and significant proteinuria with median (IQR) UPCR of 1,226 (944–1,383) mg/g. Kidney biopsy performed within 1 week of onset symptoms in all three patients with gross hematuria, significant proteinuria, and various renal function impairments revealed the presence of glomerular immune deposits predominantly stained for IgA and C3, indicative of early *de novo* IgAN (Table 1, Cases 8, 9, and 11). Steroids and/or cyclosporine were given for IgAN patients with UPCR > 2,000 mg/g and/or impaired renal function. One patient developed from AKI to advanced CKD (Case 11). Of note, the patient in Case 10 had a recurrent episode of IgAN shortly after receiving pneumococcal vaccination, whereas the patient in Case 7 experienced a recurrent episode of IgAN 6 months after contracting COVID-19 infection.

Three middle-aged MN patients consisted of two phospholipase A2 receptor antibody (PLA2R)-positive and one PLA2R-negative cases. They exhibited typical nephrotic syndrome with various degrees of AKI. Despite the immunotherapy, all MN patients had AKI transition to CKD and persistent nephrotic syndrome during the follow-up for almost 1 year (Table 1; Figure 2B). In contrast, the two middle-aged MCD patients with the typical nephrotic syndrome had achieved complete remission with intravenous and/or oral steroid treatment (Table 1).

### CKD with acute deterioration

The other 11 patients with pre-existing CKD, stage IIIa to V with median (IQR) SCr of 2.4 (1.3–3.3) mg/dL had acute deteriorated renal function without any identifiable causes such as pre-renal, post-renal, drugs, infection, poor control of hypertension, and malignancy. Their median age was 64 years (range, 46–80 years) with male-to-female ratio 6:3. Among them, four individuals had underlying diabetes mellitus, two had undergone cadaveric kidney transplantation, two had autoimmune disease (Sjogren's syndrome), two had CKD of unknown cause, and one had hypertension.

**Figure 3:**
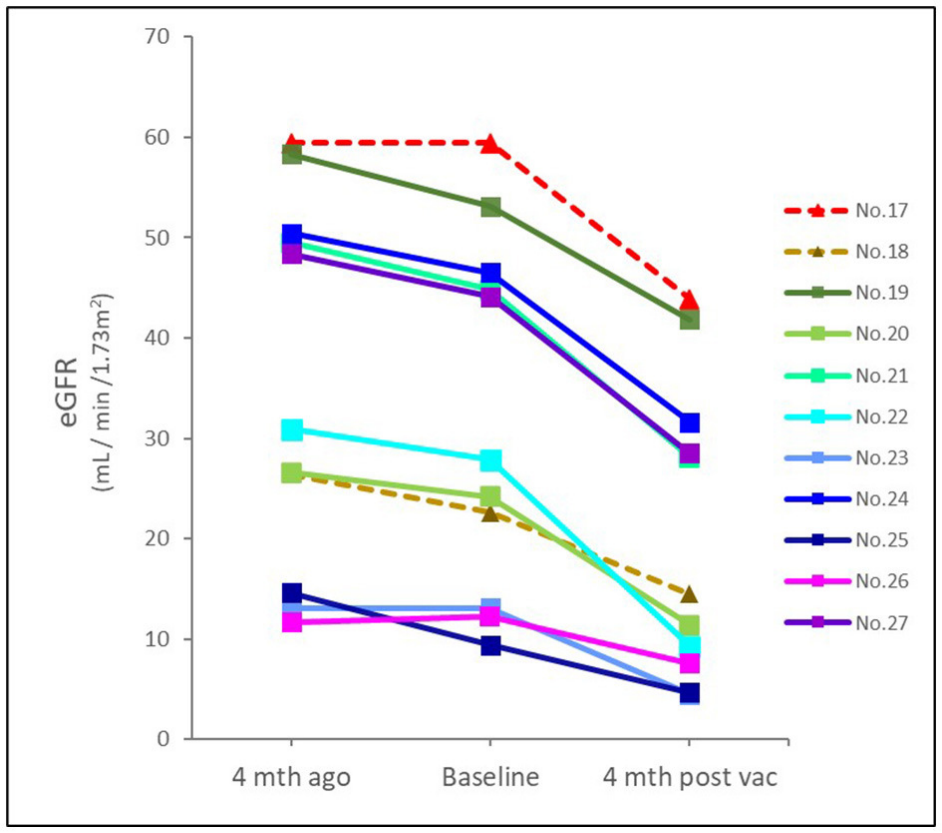
Time and eGFR change prior to and after COVID-19 vaccination in patients with CKD. The median (IQR) eGFR decline rate per 4 months prior and after vaccination was 3 (1.2–4.8) mL/min/1.73m^2^ and 10.9 (9.0–14.3) mL/min/1.73m^2^, respectively. Dotted line indicated KTR; solid line indicated non-KTR. eGFR, estimated glomerular filtration rate; mth, month; vac, vaccination.

**Figure 4:**
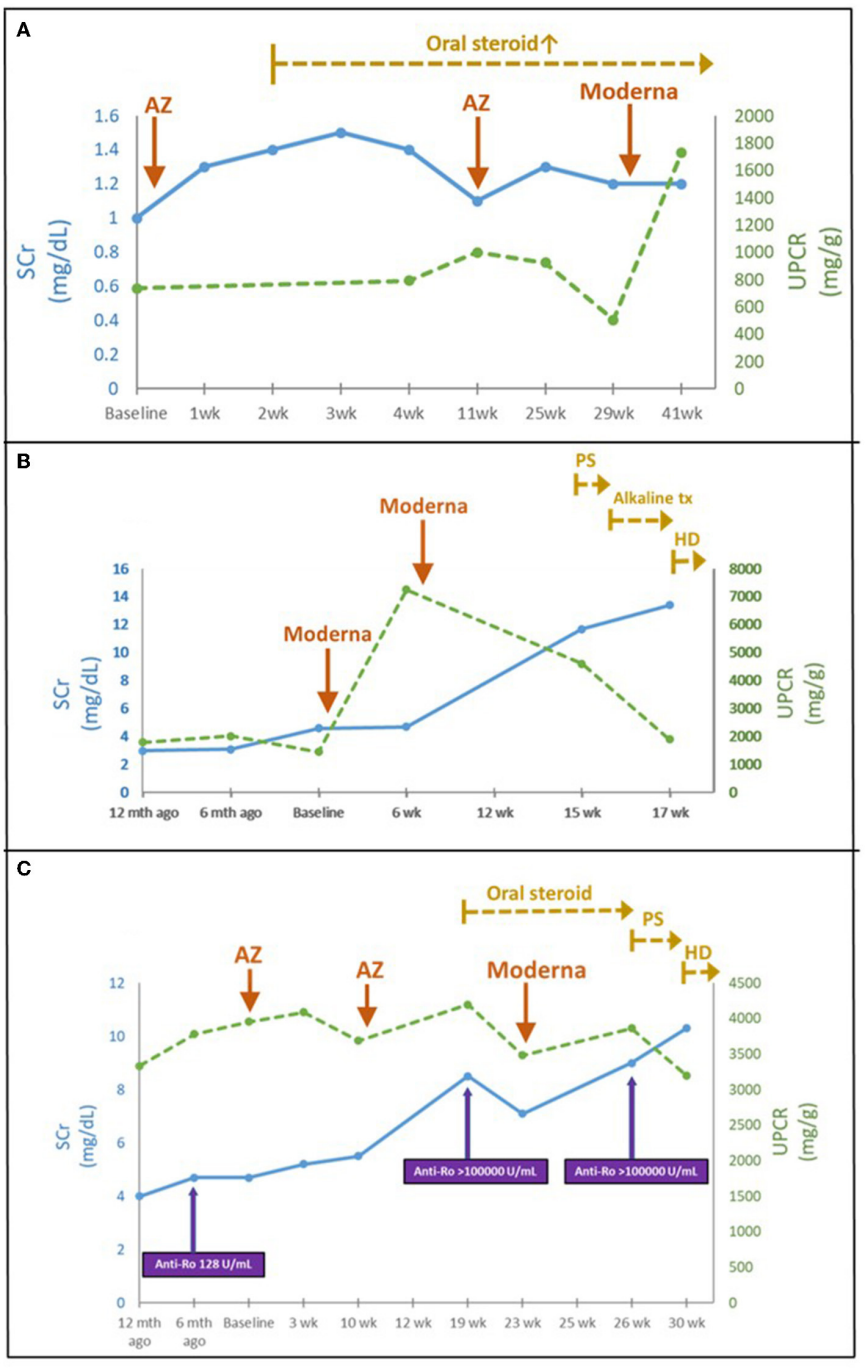
**(A)** Time course of KTR following COVID-19 vaccination (Case 17). **(B)** Acute deterioration of CKD following COVID-19 vaccination (Case 23). **(C)** Acute deterioration of CKD in a Sjogren's syndrome patient following COVID-19 vaccination (Case 25). AZ, AstraZeneca; HD, hemodialysis; Mycophenolate mofetil; P.S., pulse steroid; SCr, serum creatinine; tx, treatment; UPCR, urine protein to creatinine ratio; wk., week.

Eleven patients with CKD and acute deterioration also had a higher median (IQR) Naranjo score of 8 (7–9) despite none of them receiving further histological investigation after patient-centered shared decision-making (SDM) communication. Their median (IQR) eGFR decline rate per 4 months after vaccination was significantly accelerated from 3 (1.2–4.8) mL/min/1.73 m^2^ [over 4 (3.6–4) months] to 10.9 (9.0–14.3) mL/min/1.73 m^2^ [follow-up for 4.3 (3.9–5.6) months] (Figure 3). Edema and heavy foamy urine were the most common manifestation (9 of 11, 81.8%). One of the two kidney transplant recipients (KTRs) exhibited extra-renal features with myopericarditis in addition to declined renal function (Case 18). After the strengthened immunosuppressants and pulse steroid therapy, both KTR patients had improved renal function and proteinuria, but still had a transition from AKI to worsened CKD (Cases 17 and 18) (Table 1 and Figure 4A). Of the other nine non-KTR patients, all four patients with CKD stage III had worsened kidney function after the AKD episodes (Cases 19, 21, 24, and 27), while 75% of them (3 of 4) were with repeated COVID-19 vaccination. One diabetic patient with CKD stage III but insignificant proteinuria (SCr 1.3 mg/dL, UPCR 69 mg/g) developed AKI, KDIGO stage 3, and nephrotic syndrome (SCr 8.5 mg/dL, UPCR 11,105 mg/g) after vaccination (Case 19). He achieved improved renal function after two sessions of emergent hemodialysis (HD) and pulse steroid therapy (Table 1). One patient with Sjogren's syndrome had acute deteriorated kidney function with worsened proteinuria accompanied by a marked increase anti-Ro antibody after the repeated AstraZeneca (AZ) COVID-19 vaccination (SCr 3.7 to 8.5 mg/dL, anti-Ro antibody 128 to >100,000 U/mL), she had received emergent HD shortly after the scheduled booster dose of mRNA COVID-19 vaccination despite strengthened immunosuppressants (Case 25, Figure 4C). All five non-KTR patients with pre-existing advanced CKD has worsened to ESKD (Cases 20, 22, 23, 25, and 26), while 80% of them (4 of 5) had received multiple vaccinations (Figures 4B, C).

## Discussion

This was the first retrospective study of AKD linked to COVID-19 vaccination using the Naranjo score and peer nephrologists' review from the renal registry of a single medical center in Taiwan. An estimated rate of 13.6 per 1,000 patient-years for AKD following COVID-19 vaccination in this single-center renal registry study may be less rare than expected, as compared with an incidence rate ratio of 0.86 per 1,000,000 person-months for GN after COVID-19 mRNA vaccination from a population-based retrospective pathological cohort study (15). IgAN and *de novo* AAN were the two leading causes of GN, followed by MN and MCD. Of note, approximately 40% of them had CKD with acute deterioration characterized by worsening proteinuria and renal function without identifiable causes. Four patients exhibited extra-renal manifestations (lung and heart). Even with aggressive management, patients with *de novo* AAN and pre-existing moderate to severe CKD had poorer renal outcomes.

The COVID-19 vaccine can be viewed as a type of drug. Like any drug, COVID-19 vaccine can cause adverse drug reactions. Due to the clinical complexity involved, it remains challenging to use an empirical approach to distinguish AKD as an adverse drug reaction to the COVID-19 vaccine from other causes. Our study had a merit and novelty to first use the Naranjo score as a standard questionnaire-designed assessment for the causality of adverse reactions to the COVID-19 vaccine. Aside from temporal association, the Naranjo score gave greater weight to the recurrence of adverse events upon the re-administration of the vaccine, making it effective in assessing the impact of repeated COVID-19 vaccinations. Using the Naranjo score, we initially identified 55 of 86 patients with AKD following COVID-19 vaccination had a Naranjo score > 5 as probable or definite causality. Of note, the validity of the obtained Naranjo score should be verified by comprehensive peer reviews since the unaware confounding factors such as volume depletion, concurrent infection, inflammation, diseases progression, and other drug-related nephrotoxicity rather than COVID-19 vaccine could have easily masked intrinsic AKD. With further peer nephrologists' reviews, we excluded 28 patients with AKD caused by other causes rather than the COVID-19 vaccine.

Prior to vast COVID-19 vaccination, glomerular diseases and AKD have been reported to be temporally associated with the immunization of vaccines such as influenza, pneumococcal, and hepatitis B (23). Similarly, AKD following COVID-19 vaccination might share similar immune-mediated mechanisms to the previously reported infection, triggering subclinical disorders and manifesting them into obvious clinical diseases (24, 25). The possible mechanisms include molecular mimicry, cross-reactivity to antigens or adjuvants, and the production of certain autoantibodies (26, 27).

In the etiology of GN associated with COVID-19 vaccination, IgAN was the most common nephritic form in this study. We observed that almost all IgAN exhibited gross hematuria (within 2–3 days) after COVID-19 vaccination, similar to the rapid development of IgAN following upper respiratory tract or gastrointestinal tract infection. The post-vaccination glomerular disease mostly evaluated by the changes in eGFR and proteinuria other than hematuria in cohort studies may make IgAN largely underestimated (16, 28). As the second most nephritic form in this renal registry, all four patients with *de novo* biopsy-proven AAN also exhibited nephrotic-range proteinuria to indicate podocyte injury. Apart from a high proportion of extra-renal manifestation, their renal function rapidly progressed to either ESKD or advanced CKD yet on dialysis with persistent nephrotic-range proteinuria. Given its ominous prognosis, re-administration of COVID-19 vaccine must be evaluated with more caution in AAN patients.

Like previous reports, MCD and MN were two of the most common nephrotic syndromes after COVID-19 vaccination in our study. Both patients developed MCD after the first dose, coinciding with the possible mechanisms of rapid T cells' response to foreign mRNA by producing soluble factors, leading to podocyte dysfunction (12, 29). They were highly steroid-sensitive and did not have recurrent proteinuria after the second dose, probably related to the legacy effect of steroids. In contrast, all three patients with MN exhibited a poorer response to steroid treatment, cyclosporine, and even anti-CD20 therapy.

In CKD, both T- and B-cell dysregulation can result in reduced immune response to COVID-19 vaccination and affect the efficiency and potency of COVID-19 vaccination (30). Highly potent vaccines such as mRNA vaccines were preferred (31). Nevertheless, we still found 11 CKD patients with acute deterioration who were linked with the stronger mRNA COVID-19 vaccination. They were characterized by markedly worsened CKD and proteinuria after the exclusion of other identifiable causes. It is known that many CKD patients may have co-existing autoimmune disorders, as observed in our patients, such as Sjogren's syndrome and kidney transplant recipients under immunosuppressant therapy. These patients with acute CKD might have the potential immune and autoimmune predisposition or trigger to vaccination for renal injury. It appeared that poor steroid and/or immunotherapy responsiveness with persistent or worsening proteinuria featured in poorer kidney outcomes. Since a single episode of AKI superimposed on patients with pre-existing CKD (low renal function reserve) may cause a rapid decline in renal function, early identification of high-risk CKD patients with undiscovered autoimmune disease flaring and vulnerability to COVID-19 vaccines was needed.

There were some limitations of this study. First, the patient number was still small, while other causes of GN such as lupus nephritis or anti-glomerular basement membrane glomerulonephritis were lacking. Second, given early non-specific prodromal symptoms related to vaccination, the patients with mildly asymptomatic AKD may not be evaluated. Third, due to limited generalizability and a lack of external validity, it is challenging to apply the estimated rate of vaccine-related AKD generated by a single-center renal registry to other centers. Fourth, most COVID-19 vaccines used in Taiwan were limited to BNT162b2 (Pfizer-BioNTech), mRNA-1273 (Moderna), and AZD1222 (Oxford-AstraZeneca). As the administered vaccine types were affected by the government's policy, the risk of selection bias remains inevitable.

In conclusion, in addition to GN, AKD following COVID-19 vaccination may draw greater concern among CKD patients, particularly in those who receive repeated doses. As the COVID-19 vaccination-related AKD mainly attribute to immune dysregulation, individualized immunological evaluation and patient-centered shared decision-making (SDM) communication in patients with current or past occurrence of kidney diseases are warranted before the scheduled vaccination. Comprehensive evaluation of the adverse vaccine effects via Naranjo score and peer nephrologists' review is also recommended once AKD developed following COVID-19 vaccination. Patients with the development of *de novo* AAN, concurrent extra-renal manifestations, or pre-existing moderate to severe CKD may exhibit poorer kidney prognosis.

## Data availability statement

The original contributions presented in the study are included in the article/supplementary material, further inquiries can be directed to the corresponding author.

## Ethics statement

The studies involving human participants were reviewed and approved by Institutional Review Board (IRB) of Tri-Service General Hospital, Taipei, Taiwan (IRB No. B202205155). The patients/participants provided their written informed consent to participate in this study.

## Author contributions

C-CC and S-HL initiated and formulated the study idea and its design. S-SY, Y-JH, C-CS, PC, C-CW, S-NH, and H-EW participated in data analysis and interpretation. D-JL prepared the figures. C-CC authored the original manuscript. S-HL supervised the study. All authors contributed to the article and approved the submitted version.
